# Proteasome-driven modulation of immune and oxidative pathways during scorpion envenomation pathogenesis

**DOI:** 10.1590/1678-9199-JVATITD-2025-0007

**Published:** 2025-09-12

**Authors:** Amal Megdad-Lamraoui, Sonia Adi-Bessalem, Fares Daachi, Fatima Laraba-Djebari

**Affiliations:** 1 Laboratory of Cellular and Molecular Biology-Tamayouz, Department of Cellular and Molecular Biology, Faculty of Biological Sciences, University of Science and Technology Houari Boumediene (USTHB), Algiers, Algeria.; 2 Algerian Academy of Sciences and Technology, Algiers, Algeria.

**Keywords:** Proteasome, Scorpion venom, Cardiopulmonary tissue, Hepatorenal tissue, Immune-inflammatory response, Oxidative stress

## Abstract

**Background:**

Scorpion venom contains a variety of toxin molecules that are the drivers of inflammation and oxidative stress, leading to significant tissue damage. While several mechanisms underlying these responses have been studied, the involvement of the proteasome complex - a key regulator of inflammation - remains poorly understood. This study explored the role of the proteasome in modulating inflammatory and oxidative responses to envenomation by *Androctonus australis hector* venom.

**Methods:**

Mice were pretreated intraperitoneally with bortezomib, a proteasome inhibitor, at low (0.05 mg/kg), medium (0.25 mg/kg), or high (0.5 mg/kg) doses, 30 minutes prior to sublethal venom administration (0.5 mg/kg, subcutaneous). Twenty-four hours after venom administration, animals were euthanized, blood and organs were collected to evaluate vascular permeability (via Evans blue dye extravasation), the extent of inflammatory cell infiltration (myeloperoxidase and eosinophil peroxidase enzymatic activities), and oxidative/nitrosative stress markers (nitric oxide, hydrogen peroxide_,_ malondialdehyde, catalase activity, and glutathione). Histopathological examinations were performed to identify structural alterations, such as edema, hemorrhage, and cellular infiltration. Biochemical parameters reflecting organ function, including serum levels of CPK, LDH, ALT, ALP, urea, and creatinine, were also measured to assess the degree of systemic damage.

**Results:**

Our findings revealed a dose-dependent immune-modulatory role of the proteasome system. A medium dose of bortezomib reduced inflammatory and oxidative stress markers, such as vascular permeability, eosinophil peroxidase, neutrophil peroxidase, nitric oxide, and malondialdehyde in renal tissue, suggesting a reduction in local inflammation and oxidative damage. In contrast, a higher dose showed pronounced preventive effects in cardiopulmonary and hepatic tissues, significantly reducing inflammatory mediators and oxidative markers, restoring antioxidant enzyme activity (catalase) and glutathione, as well as, improving tissue structure and organ function.

**Conclusion:**

These findings underscore the proteasome involvement in inflammatory regulation, likely through modulation of vascular permeability, immune cell activation, and oxidative stress, making it a key target in scorpion envenomation.

## Background

Despite several scientific efforts and many therapeutic advances within the last decades, scorpion stings are still a real threat that remains among the causes of death especially in children and elderly persons in tropical and subtropical regions of the world [1-3]. Scorpion envenomation generates various signs ranging from local effects to systemic manifestations including neurotoxicity, nephrotoxicity, cytotoxicity, allergic reactions, cardiopulmonary and hepatorenal system dysfunction [4-9]. Scorpion venom contains short and long chain neurotoxic peptides that modulate the function of ion channels in neuronal terminals and are usually responsible for the main symptoms of envenoming [10, 11]. Besides these neurotoxic actions, venom components can also induce harmful inflammatory effects and excessive production of inflammatory mediators by interacting with innate immunity receptors (TLRs) and involving different inflammatory pathways [12-17].

Previous studies have demonstrated the involvement of several systems in the occurrence of inflammatory pathophysiological disturbances during scorpion envenomation - such as the adrenergic, cholinergic, histaminergic, kinin-kallikrein, renin-angiotensin-aldosterone as well as the complement system - inducing the release of different mediators including histamine, prostaglandins, leukotrienes and cytokines [6, 8, 11, 13, 16, 18-24]. Oxidative stress damage caused by excessive reactive oxygen species (ROS) is also one of the common insults encountered by cells during scorpion envenomation altering tissue integrity [25-29]. It has been shown in various types of cells that modified proteins by oxidation are selectively hydrolyzed by proteolytic systems [30, 31]. The proteasome is the main proteolytic system which is not only involved in abnormal protein degradation, it also plays a key role in several other fundamental cellular processes including the regulation of protein homeostasis, major histocompatibility (MHC) class I antigen processing, cell cycle proliferation, signaling and modulation of the immune and inflammatory responses [32-34]. 

The proteasome system is also known to be involved in regulation of the nuclear factor-κB (NF-κB) activity, which controls the transcription of a wide range of genes responsible for inflammation, such as cytokines, adhesion molecules (ICAM-1, VCAM-1), inducible nitric oxide synthase (iNOS), cyclo-oxygenase 2 (COX-2) and stress-response proteins [35-37]. 

The involvement of the proteasome system in the development of inflammation response and oxidative stress during scorpion envenomation pathogenesis has not been investigated. The overall objective of this study was to clarify the immune modulatory role of the proteasome system in peripheral tissues during scorpion envenomation.

## Methods

### Venom

Lyophilized venom of *Androctonus australis hector* (Aah) was obtained from the Biochemistry of Biomolecules: Mode of Action, Immunotherapy and Immunodiagnosis team, Laboratory of Cellular and Molecular Biology, Faculty of Biological Sciences, University of Sciences and Technology Houari Boumediene (USTHB). A previous study reported that the median lethal dose (LD₅₀) of this venom is approximately 0.85 mg/kg when administered via the intraperitoneal (i.p.) route [38]. In contrast, our prior work using subcutaneous injection - a route that closely simulates natural envenomation - demonstrated that a dose of 0.5 mg/kg induces mild envenomation without resulting in mortality [6-8, 13, 23, 24, 39].

### Chemicals and drugs

Bortezomib (Velcade^®^, PS-341) provided by Janssen-Cilag (France), is used as a proteasome inhibitor. The chemicals and reagents were mainly from Sigma (St. Louis, MO, USA) and Merck (Mannheim, Germany). 

### Animals and experimental design procedure

The experiments were performed in Algeria using Naval Medical Research Institute (NMRI) Swiss albino male mice weighing 20-22 g (7-8 weeks old) that did not undergo previous procedures. They were obtained from the animal breeding center of the Faculty of Biological Sciences, FBS-USTHB (Algiers, Algeria). They were housed under a 12-hour light/dark cycle and fed standard pellet chow and tap water *ad libitum*. All animal experiments were performed according to the Guide for the Care and Use of Laboratory Animals. All animal procedures were conducted in compliance with the ethical standards set forth by the European Parliament and Council Directive on the protection of animals used for scientific purposes (Directive 2010/63/EU). This study was approved by the Deontology and Ethic Committee of the Research Thematic Agency in Health Sciences (ATRSS) formerly National Agency of Research Development in Health (ANDRS).

In order to study the immune-modulatory role of proteasome in peripheral tissues during scorpion envenomation, the animals were randomly divided into five groups of 12 mice each. They were treated as follows: the first group serving as control received an injection of 100 μL/mouse of physiological saline water (NaCl) at 0.9% by subcutaneous route (s.c). The second group represents envenomed mice with a sublethal dose at 0.5 mg/kg (s.c.), while the third, the fourth, the fifth and the sixth groups consist of mice receiving three doses of bortezomib (0.05, 0.25, 0.5 mg/kg) tested intraperitoneally. About 30 minutes later, mice were envenomed with a sublethal dose of Aah venom (0.5 mg/kg, s.c.). Control and envenomed mice were euthanized 24 hours after the injection of physiological saline or venom. In addition to blood, the heart, lungs, liver, kidneys (from control or treated animals with venom at 0.5 mg/kg) were collected 24 hours after NaCl or venom injection, weighed, and then used for further investigations. 

### Enumeration of the different peripheral blood leukocyte populations

Blood samples were taken in tubes with EDTA 24 hours after the injection of Aah venom. The cell count was carried out by a hemocytometer ERMA INC (full automatic blood cell counter model PCE-210N) analyzer. Blood cells were identified according to the position of the nucleus, its area and its density. The results are expressed as 10^3^ cells/μL of blood.

### Serum protein electrophoresis 

Serum protein electrophoresis was carried out using an automatic device of the “Capillary SEBIA” type. Serum proteins (albumin and α1, α2, β, γ globulins) are amphoteric molecules, their separation takes place according to their electrical charges. The serum samples from the different groups were placed in a basic medium (cellulose acetate) where the serum proteins acquired an overall negative charge allowing them to migrate from the cathode to the anode under the influence of an electric field. The migration was stopped as soon as the separation was sufficient; a fixation was then carried out with the acid blue dye. Five zones corresponding to the different serum proteins having decreasing negative charges were obtained. The results were expressed in g/L. 

### Vascular permeability and inflammatory cell infiltration

*Vascular permeability*

To measure changes in vascular permeability, Evans blue dye was administered intravenously (20 mg/kg) immediately before the injection of NaCl or venom [40]. The organs - heart, lungs, liver and kidneys - were collected after mouse euthanasia. They were then placed in formamide for the extraction of Evans blue, then incubated at 37°C for 72 hours. Absorbance was read at 620 nm and the results were expressed as the concentration of Evans blue per µg of tissue.

*Myeloperoxidase activity*

The accumulation and activation of neutrophils were analyzed by measuring myeloperoxidase (MPO) in tissue homogenates according to the method described by Coelho et al. [41]. The oxidation of the chromogenic substrate, O-dianisidine, by MPO was measured by spectrophotometry at 460 nm. A volume of 1 mL of 50 Mm phosphate buffer (pH 6.6) containing O-dianisidine dihydrochloride (0.167 mg/mL) and hydrogen peroxide, was added to 1 mL of biological sample. The oxidation of the chromogenic substrate O-dianisidine by MPO was measured spectrophotometrically at 460 nm Changes in absorbance were recorded over 2minutes. MPO activity was expressed as mM/min/100 mg of tissue or mM/min/ mL of serum, using an extinction coefficient (ε) of 11.3 mM⁻¹·cm⁻¹.

*Eosinophil peroxidase activity*

The evaluation of the eosinophil peroxidase (EPO) activity, a marker for eosinophil accumulation, was carried out according to the method described by Van Oosterhout et al. [42]. Supernatants (50 µL) were placed in the wells of a microplate with 100 μL of buffer solution [50 mM Tris-HCl, pH 8, containing 20 mg of o-phenylenediamine (OPD) and 10 µL of H₂O₂]. Enzyme activity was assessed by measuring absorbance at 490 nm after 1 hour of incubation at 37°C. Results are expressed as absorbance per 100 mg of tissue.

### Oxidative stress markers

Oxidative stress was evaluated by measuring the levels of pro-oxidant (malondialdehyde and nitrites) and antioxidants biomarkers (catalase CAT and glutathione GSH) in tissue homogenates.

*Nitric oxide*

The level of nitric oxide was determined by measuring nitrites [43]. Nitrite levels were evaluated in the various samples using the Griess method. The samples were deproteinized with TCA (10%) and then incubated volume for volume with Griess reagent (1% sulfanilamide and 0.1% naphthylethylenediamine dihydrochloride in 2.5% phosphoric acid) for 20 min at room temperature. Nitrite levels were measured spectrophotometrically at 540 nm. A standard curve was prepared using a stock solution of KNO_2_. Nitrite concentrations are expressed in µM/g of tissue.

*Hydrogen peroxide*

The estimation of hydrogen peroxide (H_2_O_2_) levels is based on the oxidation of phenol red by H_2_O_2_ via peroxidase [44]. The samples were distributed in a microplate at a rate of 100 µL/well supplemented with 100 µL of a reactive solution of phenol red [0.01 g glucose, 0.0001 g of horseradish peroxidase (HRP) 0.0001 g phenol red in 10 mL PBS]. The plate was then incubated for one hour at 37°C, protected from light. The reaction was stopped by adding 10 µL of NaOH (1N). Absorbance readings were taken at 620 nm microplate reader. The concentration of hydrogen peroxide was determined after extrapolation on a standard curve made for different concentrations of H_2_O_2_ ranging from 0.005 mM to 0.500 mM. Results were expressed as mM/100 mg tissue.

*Malondialdehyde*

Malondialdehyde, an indicator of lipid peroxidation, is one of the end products of the breakdown of polyunsaturated fatty acids, it is released under the effect of free radicals during stress. The malondialdehyde level was evaluated according to the method of Moreno et al. [45]. The samples from the different groups are incubated (v/v) with TCA (35 %) for 1 hour at 4°C for protein precipitation. After centrifugation of the mixture for 10 minutes at 4000 *g*, 200 μL of the supernatant were mixed with 100 μL of SDS (8.1%), 750 μL of acetic acid (20%), 750 μL of thiobarbituric acid (0.8%) and 200 μL of distilled water. The mixture was then incubated at 100°C for 1 hour, followed by cooling in ice. The optical density was measured at 532 nm and the amount of malondialdehyde formed was calculated using a molar extinction coefficient of 1.56 x 10^5^ M^-1^ cm^-1^. Results were expressed in nM/ 100 mg of tissue.

*Catalase activity*

Catalase is a ubiquitous antioxidant enzyme. It is responsible for the conversion of hydrogen peroxide (H_2_O_2_) into water (H_2_O) and oxygen (O_2_). The enzymatic reaction is triggered by the addition of 50 μL of each sample and of the substrate (0.2% H_2_O_2_) in phosphate buffer (50 mM at pH 7). The kinetics of H_2_O_2_ degradation were measured spectrophotometrically at 240 nm for 3 min. One unit of catalase is equal to (2.3/T) (Log A1/A2), where T is the time interval in minutes, A1 the absorbance in the first minute and A2 the absorbance in the second minute [46]. Catalase activity was expressed in U/100 mg of tissue.

*Glutathione*

Glutathione plays an important role in the detoxification and reduction of both organic and inorganic peroxides, in the presence of glutathione peroxidase (GPx). Its quantification is based on the measurement of the optical absorbance of 2-nitro-5-mercapturic acid, which results from the reduction of 5,5'-dithio-bis-2-nitrobenzoic acid by the (-SH) groups of GSH [47]. To carry out this assay, a volume of 50 µL of sample was mixed with 100 µL of DTNB (5,5'-dithio-bis (2-nitrobenzoic acid) and 100 µL of phosphate buffer (0.1 M and pH 7.4) The absorbance reading was taken at 450 nm after a 30 minute incubation at 37°C. The results were expressed in µM / 100 mg of tissue using a molar extinction coefficient of 13.3 M^-1^.cm^-1^

### Histopathological analysis

The effects of proteasome inhibitor on Aah venom induced cardiac, pulmonary, and hepatorenal tissues alteration were examined by histological analysis. 

Organs (liver, kidneys, heart, lungs) were collected from the animals, fixed in formalin (10%), and embedded in paraffin. The resulting tissue blocks were sectioned using a microtome to obtain 5 µm thick sections. These sections were stained with hematoxylin and eosin to visualize tissue damage under a light microscope.

### Evaluation of organ functions

Organ function was assessed by estimating the enzyme activities of creatine phosphokinase (CPK), lactate dehydrogenase (LDH), alanine aminotransferase (ALT), alkaline phosphatase (ALP), as well as by measuring urea and creatinine levels in serum, according to the manufacturer's (Spinreact, Spain) instructions. Enzyme activities were expressed in international units (IU/L). Serum creatinine and urea levels were expressed in g/L.

### Statistical analysis 

Results are expressed as means ± SEM. Statistical comparisons were carried out using One-Way ANOVA (Graph Pad Prism 5 Software, San Diego, CA, USA) to identify differences between the envenomed and control groups or between the pretreated and the envenomed groups. A value of *p* < 0.05 was considered statistically significant.

## Results

### Effects of proteasome inhibitor on blood cells and serum alpha, beta and gamma globulins concentration

**Figure 1.**
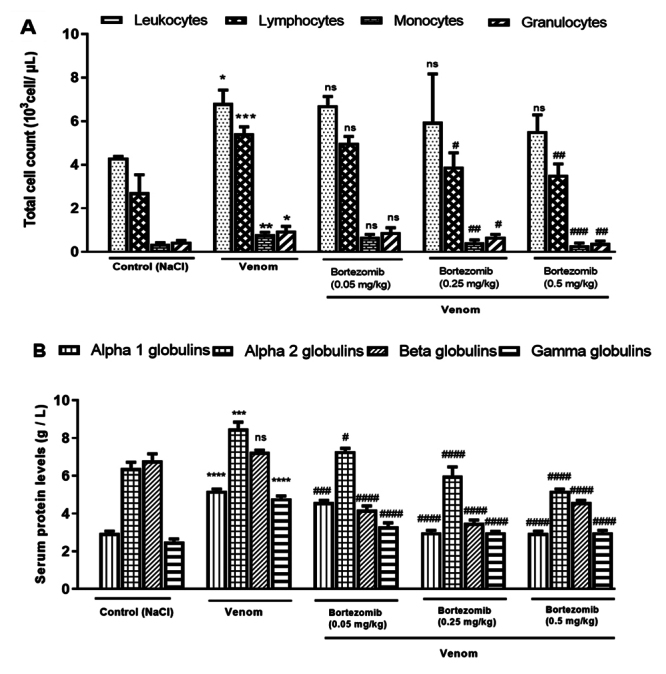
(A) Effects of proteasome inhibition on leukocyte levels and (B) serum protein contents 24 hours after envenomation. Values are expressed as mean ± SEM (n = 3/group). Student's t-test, *p < 0.05; **p < 0.01; ***p < 0.001; ****p <0.0001: groups of envenomed (0.5 mg/kg) mice compared to the control group (NaCl). ^#^p < 0.05; ^##^p < 0.01; ^###^p < 0.001; ^####^p <0.0001: pretreated groups compared to the group of envenomed (0.5 mg/kg) mice. ns: non-significant results.

Blood leukocyte levels were assessed in the presence of low (0.05 mg/kg), medium (0.25 mg/kg), or high (0.5 mg/kg) doses of proteasome inhibitor before experimental envenomation. The results showed that scorpion venom induced hyperleukocytosis 24 hours after envenomation of the mice, compared to the control group, as evidenced by a significant increase in the levels of lymphocytes (*p* < 0.001), monocytes (*p* < 0.01), and granulocytes (*p* < 0.05) (Figure 1A). This migration is likely induced by various immune stimuli, such as the pro-inflammatory cytokines released after scorpion envenomation [48-51]. 

Serum proteins from the acute phase of inflammation are key markers of the inflammatory response. Electrophoresis analysis revealed an increase in the alpha and gamma globulin fractions in the sera of envenomed mice exposed to a sublethal dose of Aah venom (Figure 1B). Gamma globulins are synthesized following the activation of B lymphocytes. The increase in this fraction indicates the synthesis of various types of immunoglobulins in serum (IgA, IgG, IgM, IgD, IgE) and C-reactive protein (CRP).

Higher doses of proteasome inhibitor (0.25 and 0.5 mg/kg) induced a significant decrease in the level of lymphocytes (*p* < 0.05 and *p* < 0.01), monocytes (*p* < 0.01 and *p* < 0.001) and granulocytes (*p* < 0.05 and *p* < 0.01), comparatively to that of envenomed mice. It seems that this pretreatment was able to significantly prevent hyperleukocytosis induced by Aah venom in the blood (Figure 1A).

With regard to the effects on the serum protein electrophoresis, pretreatment of mice with a low dose of bortezomib (0.05 mg/kg) induced a significant decrease in all globulin fractions (α1, α2, β, and γ) compared to the values recorded in the envenomed group of mice. Bortezomib at 0.25 and 0.5 mg/kg significantly reduced the α1, α2, β- and γ-globulin fractions, compared to the envenomed group of mice. However, a more pronounced decrease in all globulin fractions was observed at the higher concentration of bortezomib (0.5 mg/kg), compared to the effects recorded at the lower doses (Figure 1B).

### Effect of pretreatment with proteasome inhibitor on serum albumin level and organ water content

**Figure 2.**
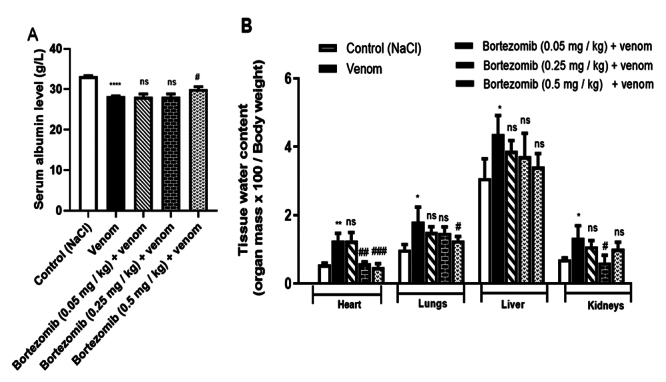
**(A)** Effects of proteasome inhibition on serum albumin levels and **(B)** peripheral tissue water content 24 hours after envenomation. Values are expressed as mean ± SEM (n = 3/group). Student's t-test, *p < 0.05; **p < 0.01; ***p < 0.001; ****p < 0.0001: groups of envenomed (0.5 mg/kg) mice compared to the control group (NaCl). ^#^p < 0.05; ^##^p < 0.01; ^###^p < 0.001; ^####^p < 0.0001: pretreated groups compared to the group of envenomed (0.5 mg/kg) mice. ns: non-significant results.

Compared with the control group, the levels of albumin were significantly decreased in envenomed animals (*p* < 0.0001) (Figure 2A). This hypoalbuminemia is accompanied by high water content in the heart, lungs, liver and kidneys of envenomed animals compared with normal controls. Albumin is a negative acute phase protein. Leakage of serum albumin levels due to increased vascular permeability can contribute to edema formation in tissues [52]. Bortezomib at 0.5 mg/kg significantly prevented the decrease in albumin rates compared with the venom group (*p* < 0.05). However, no significant effect was observed at lower concentrations (0.05 and 0.25 mg/kg) (Figure 2A). Similarly, with regard to tissue water content, bortezomib at the highest concentration tended to prevent edema formation in the cardiac, pulmonary, kidneys and hepatic tissues (Figure 2B). These results suggest that Aah venom induces edema formation, by activating in part the proteasome complex.

### Evaluation of vascular permeability and inflammatory cell infiltration in peripheral tissues

**Figure 3.**
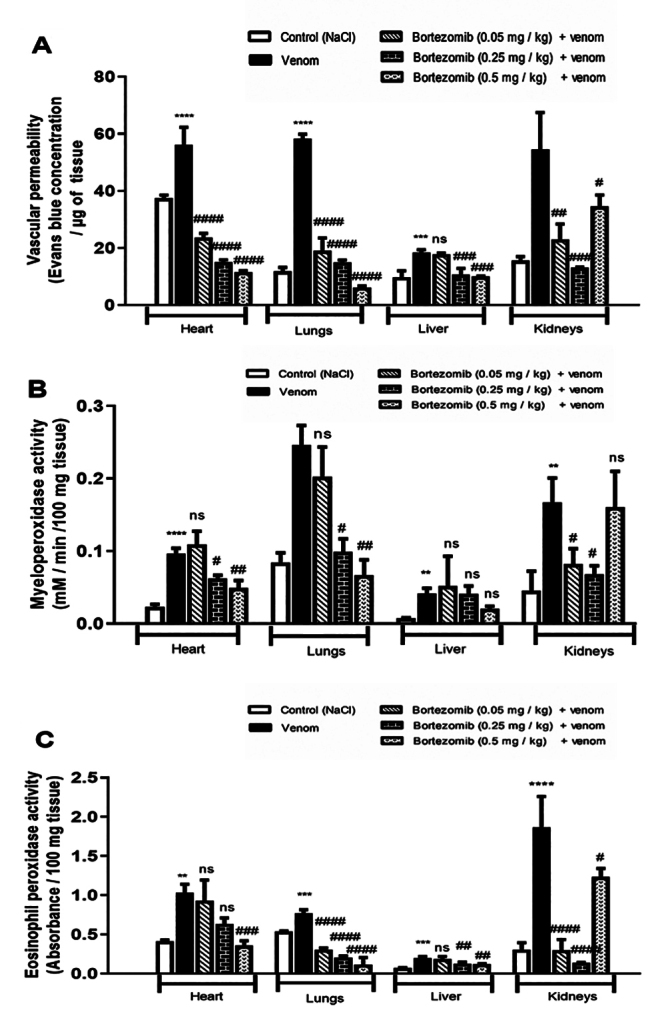
Effects of low (0.05 mg/kg), medium (0.25 mg.kg) or high dose (0.5 mg/kg) of bortezomib on **(A)** vascular permeability changes and inflammatory cell infiltration including **(B)** neutrophils and **(C)** eosinophils in peripheral tissues of envenomed mice by Aah venom. Values are expressed as mean ± SEM (n = 3/group). Student's *t*-test, *p < 0.05; **p < 0.01; ***p < 0.001; ****p < 0.0001: groups of envenomed mice compared to the control group (NaCl). ^#^p < 0.05; ^##^p < 0.01; ^###^p < 0.001; ^####^p < 0.0001: pretreated groups compared to the group of envenomed (0.5 mg/kg) mice. ns: non-significant results.

Vascular permeability in various peripheral tissues was assessed by quantifying Evans blue extravasation [40]. The increase in vascular permeability in damaged tissue reflects the movement of a significant volume of plasma from blood capillaries into the tissues. This plasma flow transports a substantial number of inflammatory mediators [53] leading in particular to edema formation in tissues. Results showed that 24 hours after injection, Aah venom induces a significant increase in vascular permeability of heart (*p* < 0.0001), lungs (*p* < 0.0001), liver (*p* < 0.001) and renal (*p* < 0.001) tissues (Figure 3A). These results are similar to those carried out previously by our team [6-8, 29]. 

The increase in vascular permeability facilitates the migration of leukocytes from the vascular system to the injured area. Peroxidase activities were measured as markers of neutrophil and eosinophil activation. Myeloperoxidase and eosinophil peroxidase activities were significantly elevated in the tissue homogenates of animals injected with Aah venom compared to the control group (Figure 3B and 3C). 

According to the results, pretreatment with low (0.05 mg/kg), medium (0.25 mg/kg), and high (0.5 mg/kg) doses of proteasome inhibitor significantly reduced Aah venom-induced Evans blue extravasation in cardiac and pulmonary tissues in a concentration-dependent manner. However, in renal tissue, this effect was not dose-dependent, as the highest dose of bortezomib (0.5 mg/kg) failed to prevent the increase in vascular permeability to the same extent as the lower dose (Figure 3A).

The activities of myeloperoxidase and eosinophil peroxidase significantly decreased in the group of animals treated with a higher dose of bortezomib (*p* < 0.001, *p* < 0.0001, *p* < 0.01) compared to the values observed in the heart, lungs, and liver tissues of animals envenomed with Aah venom alone. In the renal tissue it appears that, the medium dose (0.25 mg/kg) of bortezomib is more effective (*p* < 0.05) than the low and high doses in reducing the infiltration of neutrophils and eosinophils (Figure 3B and 3C).

### Evaluation of oxidative stress status in peripheral tissues in the presence or absence of increasing doses of proteasome inhibitor

**Figure 4.**
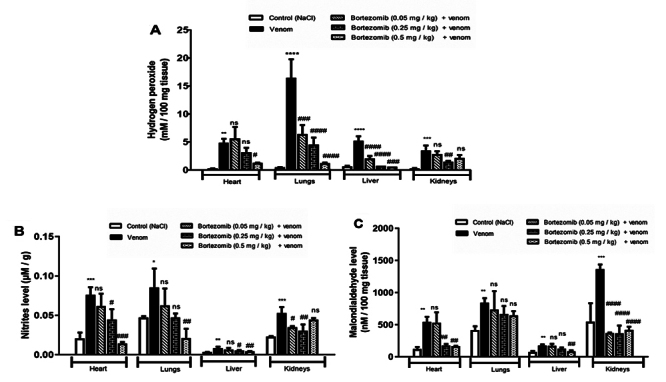
Effects of low (0.05 mg/kg), medium (0.25 mg.kg) or high dose (0.5 mg /kg) of bortezomib on **(A)** hydrogen peroxide, **(B)** nitrites and **(C)** malondialdehyde levels in peripheral tissues of envenomed mice by Aah venom. Values are expressed as mean ± SEM (n = 3/group). Student's *t*-test, *p < 0.05; **p < 0.01; ***p < 0.001; ****p < 0.0001: groups of envenomed (0.5 mg/kg) mice compared to the control group (NaCl). ^#^ *p* < 0.05; ^##^ *p* < 0.01; ^###^ *p* < 0.001; ^####^ *p* < 0.0001: pretreated groups compared to the group of envenomed (0.5 mg/kg) mice. ns: non-significant results.

The inflammatory response induced by Aah venom was associated with a significant increase in nitrites, hydrogen peroxide, and malondialdehyde levels, along with a decrease in glutathione content and catalase activity compared to the controls (Figures 4A, 4B and 4C). Previous studies have suggested that the infiltration of inflammatory cells (including neutrophils and eosinophils) may lead to increased release of reactive oxygen and nitrogen species accompanied by an alteration of the antioxidant defense system [6, 54]. 

Pretreatment with bortezomib reduced the levels of pro-oxidant markers (MDA, nitrites, H_2_O_2_) and prevented dysfunction of the antioxidant defense system (CAT and GSH) in the peripheral tissue homogenates of envenomed mice. The high dose (0.5 mg/kg) appears to be more effective in reducing nitrite and hydrogen peroxide levels, as well as lipid peroxidation, compared to the low and medium doses in all studied tissues, except in renal tissue, when compared to the respective values recorded in envenomed mice without pretreatment (Figures 4A, 4B and 4C). In the renal tissue, significant decrease in pro-oxidant markers were noted in the group receiving a medium-dose (0.25 mg/kg) of bortezomib in comparison with envenomed mice (Figures 4A, 4Band 4C). 

**Figure 5.**
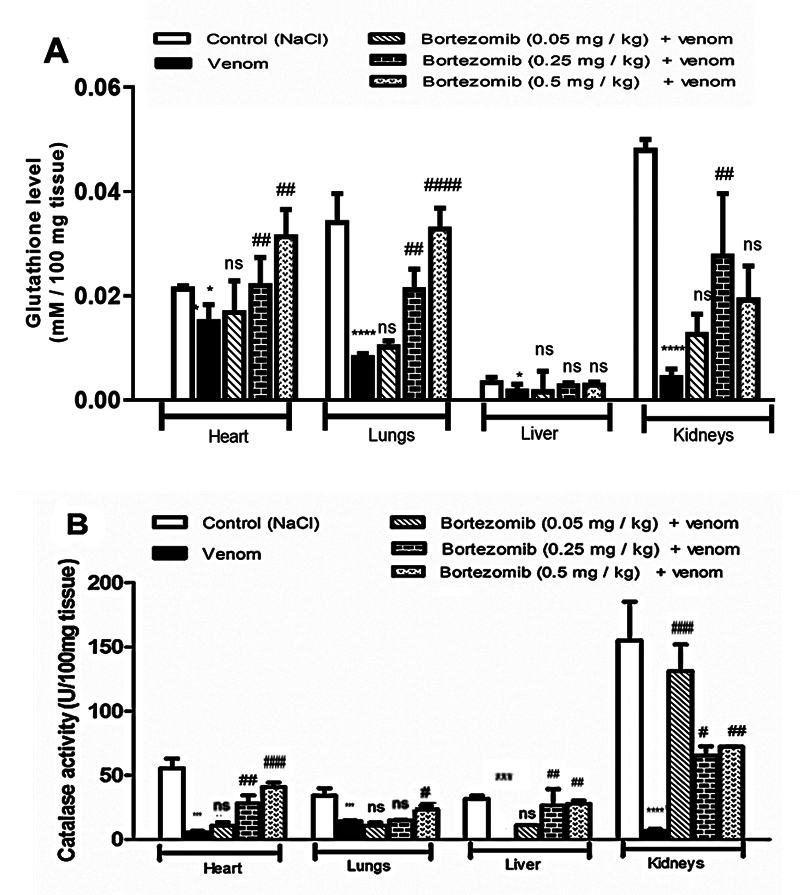
Effects of bortezomib (0.05 mg/kg, 0.25 mg.kg and 0.5 mg /kg) on **(A)** glutathione and **(B)** catalase activity in peripheral tissues of envenomed mice by Aah venom. Values are expressed as mean ± SEM (n = 3/group). Student's *t*-test, *p < 0.05; **p < 0.01; ***p < 0.001; ****p < 0.0001: groups of envenomed (0.5 mg/kg) mice compared to the control group (NaCl). ^#^ *p* < 0.05; ^##^ *p* < 0.01; ^###^ *p* < 0.001; ^####^ *p* < 0.0001: pretreated groups compared to the group of envenomed (0.5 mg/kg) mice. ns: non-significant results.

The decrease in pro-oxidant markers was associated with an improved antioxidant status, characterized by the restoration of catalase activity and GSH levels in peripheral tissues, compared to the animals injected with Aah venom. Significant results were observed in heart and lung homogenates of mice pretreated with the high dose of bortezomib. At the hepatic level, this dose did not have an effect on the restoration of glutathione levels in the liver. In the kidneys, it appears that the medium and low doses were respectively more effective in preventing alterations in the non-enzymatic (*p* > 0.05, *p* < 0.001) and enzymatic (*p* < 0.05) antioxidant systems (*p* < 0.0001, *p* < 0.05) and enzymatic (*p* < 0.05) (Figure 5A and 5B).

### Histopathological analysis and the effects of proteasome inhibitor on biomarker levels of organ function in envenomed animals

**Figure 6.**
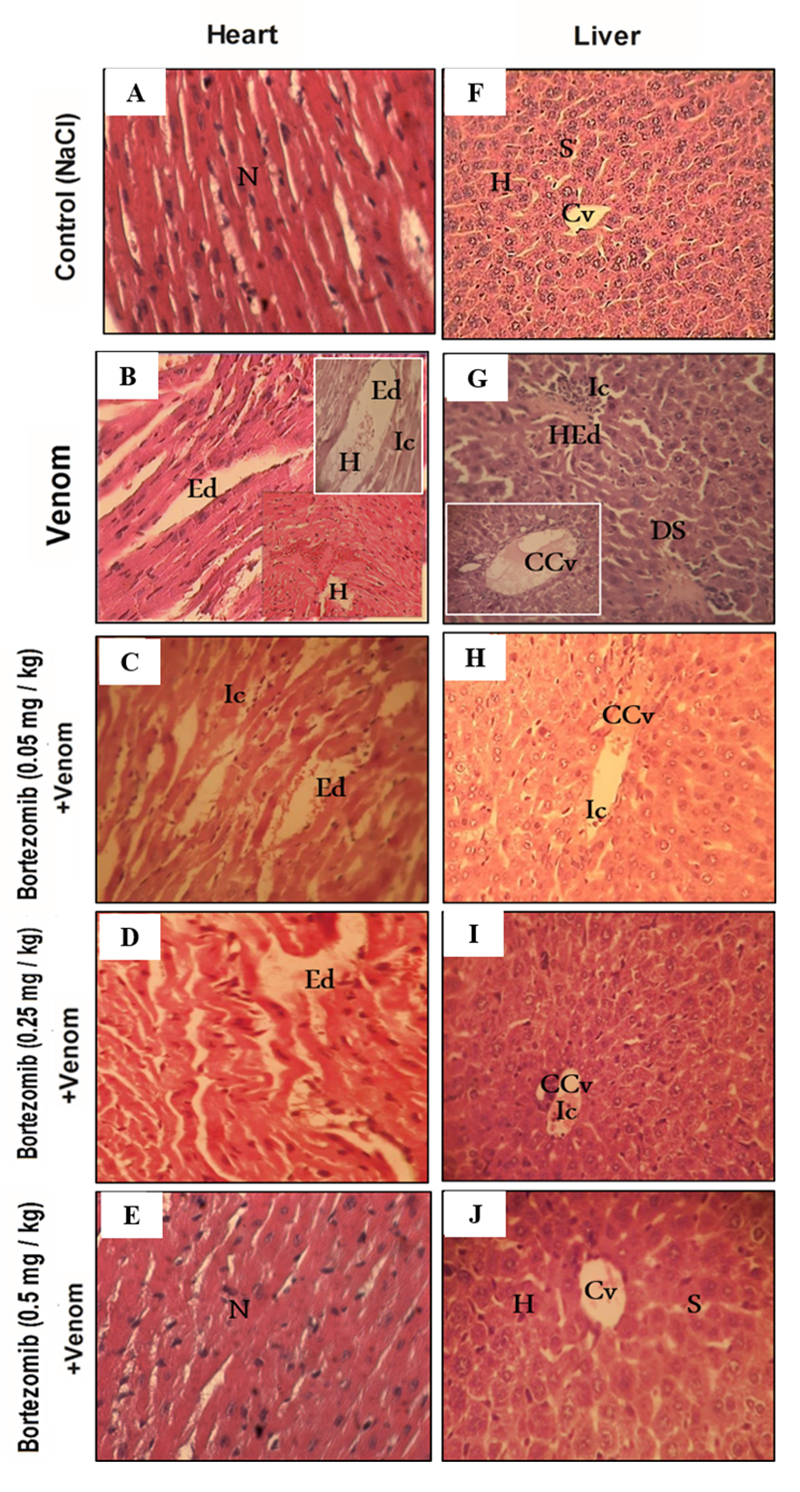
Effects of proteasome inhibition on (**A-E**) myocardial and (**F-J**) hepatic tissue, 24 h after Aah envenomation. (**A, F**) Control (NaCl). (**B, G**) Animals injected with Aah venom (s.c). (**C, H**) Animals pretreated with a low dose bortezomib (0.05 mg/kg, i.p.). (**D, I**) Animals pretreated with a medium dose bortezomib (0.25 mg/kg, i.p.). (**E, J**) Animals pretreated with a high dose bortezomib (0.5 mg/kg, i.p.). Hematoxylin-eosin staining, magnification × 400. Cv: center-lobe vein; CCv: congested center-lobe vein; Ed: edema; H: hemorrhage; HEd: hemorrhagic edema; Ic: inflammatory cell infiltrates; N: nucleus.

**Figure 7.**
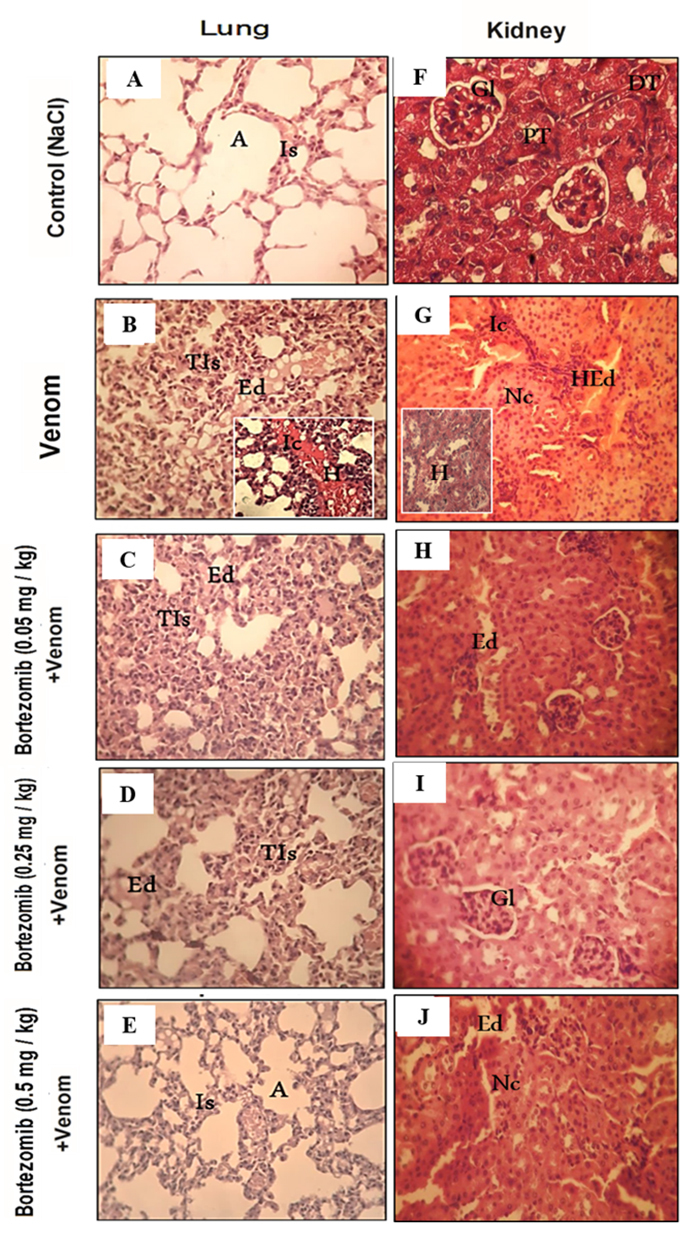
Effects of proteasome inhibition on (**A-E**) pulmonary and (**F-J**) renal tissue structure, 24 h after Aah envenomation. (**A, F**) Control (NaCl). (**B, G**) Animals injected with Aah venom (s.c). (**C, H**) Animals pretreated with a low dose bortezomib (0.05 mg/kg, i.p.). (**D, I**) Animals pretreated with a medium dose bortezomib (0.25 mg/kg, i.p.). (**E, J**) Animals pretreated with a high dose bortezomib (0.5 mg/kg, i.p.). Hematoxylin-eosin staining, magnification × 400. A: alveolar; DT: distal tubule; E: edema; Gl: glomerulus; HEd: hemorrhagic edema; H: hemorrhage; Ic: inflammatory cell infiltrates; Is: inter-alveolar septum; Nc: necrosis; PT: proximal tubule; TIs: thick inter-alveolar septum.

To further evaluate the role of the proteasome in tissue injuries induced by scorpion venom, histopathological analysis of H&E-stained sections was performed. Histological sections of heart, liver, lung, and kidney tissues from the control group showed normal structure without any histological changes (Figures 6A and 6F, and Figures 7A and 7F). However, scorpion venom administration induced tissue damage, characterized by the loss of normal histological structure, infiltration of inflammatory cells, thickening of the alveolar septa, as well as edema and hemorrhage (Figures 6Band 6G, and Figures 7B and 7G). These histological changes were accompanied by a significant increase in CPK, ALT, ALP, LDH, urea, and creatinine levels in the sera of envenomed mice (Table 1). The increase in these markers may be attributed to impaired organ function, as shown in previous studies [55, 56]. 

Following treatment with a high dose of bortezomib, administered intraperitoneally 30 minutes before envenomation, tissue alterations were significantly reduced in the heart, liver, and lungs (Figures 6E and 6J, and Figure 7E). This pretreatment significantly reduced the activities of CPK (69.28%), ALT (23.63%), ALP (52.37%), and LDH (20.67%) compared to the animals injected with Aah venom (Table 1). The low and medium doses of bortezomib treatment resulted in a slight reduction in histological injuries in these organs. However, some histological alterations, such as edema, inflammatory cell infiltration, and congestion of the central vein, persisted (Figures 6C, 6H, 6D and 6I, and Figures 7Cand 7D). 

In the renal tissue, marked reduction of tissue alterations was observed in average-dose (0.25 mg/kg) of bortezomib in pretreated group of mice (Figure 7I) accompanied with significant decrease in urea (73.21%) and creatinine (28.42%) levels when compared to envenomed group of mice (Table 1). 

**Table 1.** Effects of proteasome inhibition on serum biomarkers of tissue damage. Values represented in the table are expressed as mean ± SEM (n = 3/group). Student's *t*-test, *p < 0.05; **p < 0.01; ***p < 0.001; ****p < 0.0001: groups of envenomed (0.5 mg/kg) mice compared to the control group (NaCl). ^#^ *p* < 0.05; ^##^ *p* < 0.01; ^###^ *p* < 0.001; ^####^ *p* < 0.0001: pretreated groups compared to the group of envenomed (0.5 mg/kg) mice. ns: non-significant results.

	Control (NaCl)	Venom	Bortezomib (0.05 mg/kg) + venom	Bortezomib (0.25 mg/kg) + venom	Bortezomib (0.5 mg/kg) + venom
**CPK (IU/L)**	1626.66 ± 46.45	2530 ± 107.33^***^	2070.33 ± 306.77^#^	2000.33 ± 100.10^#^	1605.66 ± 2.88^###^
**ALT (IU/L)**	35.83 ± 4.59	170.28 ± 18.25^****^	164.16 ± 7.63 ^ns^	132.00 ± 12.58 ^#^	38.33 ± 12.58^####^
**ALP (IU/L)**	84.53 ± 9.47	193.89 ± 17.68^**^	122 ± 31.32^#^	100 ± 21.32^##^	102.33 ± 28.29^####^
**LDH (IU/L)**	1698.66 ± 34.42	1895.00 ± 107.58	1836.68±60.27^ns^	1721.66 ± 235.39^ns^	1503.33 ± 66.16^#^
**Urea (g/L)**	0.16 ± 0.04	0.42 ± 0.03^****^	0.40 ± 0.01^ns^	0.26 ± 0.01^##^	0.37 ± 0.06^ns^
**Creatinine (g/L)**	3.33 ± 0.57	11.33 ± 2.3^**^	8 ± 2.00 ^ns^	7 ± 2.00^ns^	11 ± 2.64^ns^

## Discussion 

Involvement of the proteasome system during various inflammatory diseases was reported and has been directly linked to the progression of these diseases [57-61]. However, no study has yet demonstrated its role in the pathogenesis of scorpion envenomation. 

In this study, we investigated the role of the proteasome in Aah venom-induced toxicity and evaluated the potential immune-modulatory role of bortezomib, a proteasome inhibitor, through its impact on vascular, oxidative and biochemical markers.

The present study showed that experimental envenomation with Aah venom triggered systemic immune system activation, characterized by increased blood cell counts and acute-phase protein levels, along with hypoalbuminemia. These findings are consistent with previous studies on scorpion envenomation, which have also reported systemic inflammatory responses, including leukocytosis, elevated acute-phase reactants, and reduced albumin levels [62-64]. The earliest response to envenomation is vascular dysfunction, characterized by increased vascular permeability and tissue edema. This was reflected in our study by elevated tissue water content and Evans blue dye extravasation in multiple organs. Similar results were reported in previous studies on scorpion envenomation [6, 8]. Hypoalbuminemia observed in this context may result from increased vascular permeability, which facilitates the leakage of albumin and other plasma proteins into tissues. Hypoalbuminemia reduces plasma oncotic pressure, leading to fluid leakage into tissues, which results in organ water accumulation, tissue swelling, and potential functional impairment [65]. The vascular leakage is often associated with inflammatory cell infiltration, as confirmed in this study by the elevated activities of myeloperoxidase (MPO) and eosinophil peroxidase (EPO), enzymatic markers of neutrophil and eosinophil accumulation, respectively. These immune cells are known to be major sources of reactive oxygen species, such as hydrogen peroxide (H₂O₂) generated from superoxide anions, as well as reactive nitrogen species like nitric oxide (NO) [66, 67]. The overproduction of nitric oxide (NO) and reactive oxygen species (ROS) during envenomation leads to a significant redox imbalance, partly due to reduced antioxidant defense, as shown by the decreased glutathione level and catalase activity likely inhibited by excess H₂O₂ [68, 69]. These reactive species act as cytotoxic agents by inducing lipid peroxidation, compromising cell membrane integrity [70]. This was evaluated by elevated malondialdehyde (MDA) levels, both in our study and in previous experimental and clinical reports [48, 71], reflecting oxidative tissue damage. Histological analysis further revealed venom-induced cardiac, pulmonary and hepatorenal alterations, including hemorrhage, edema, and inflammatory cell infiltration. These results were also obtained by previous studies on scorpion envenomation [4, 6, 13, 17, 23, 24, 72-74]. 

The histological alterations induced by Aah venom were accurately reflected in the serum by elevated CPK, ALT, ALP and LDH activities, which are commonly associated with the severity of tissue damage in cases of scorpion envenomation [6, 19]. These pathological manifestations are consistent with clinical observations in human scorpion envenomation**.** They include systemic inflammatory response, cardiovascular dysfunction, pulmonary edema [75-77] and hepatorenal manifestations [78-80]. Several studies have emphasized the relevance of pro-inflammatory mediators in the pathophysiological manifestations of human scorpion envenomation [16]. Moreover, there is a significant association between these complications and oxidative stress induced by venom components [81], as well as the release of organ dysfunction biomarkers such as LDH, CPK, and ALT, which are commonly elevated in clinical cases of envenomation and reflect systemic tissue injury [64, 78].The findings of the present study are in line with these clinical features, as experimental envenomation in mice led to increased vascular permeability, inflammatory infiltration, and organ damage. The observed increase in inflammatory and oxidative markers mirrors the pathophysiological processes seen in human victims, thereby validating the experimental model used.

The results of this study demonstrated that pretreatment of mice with a proteasome inhibitor introduced at low (0.05 mg/kg), medium (0.25 mg/kg) or high (0.5 mg/kg) doses prior to administration of the venom reduced the levels of inflammatory and oxidative markers seen in scorpion envenomation in a dose-dependent manner. A more preventive effect was observed at the cardiac, pulmonary and hepatic levels in the presence of a high concentration of bortezomib (0.5 mg/kg). In the renal tissue, it appears that the medium dose (0.25 mg/kg) of the proteasome inhibitor is more effective in reducing the immune inflammatory response. Several studies have demonstrated the effectiveness of these doses in reducing inflammation response during experimental inflammatory pathologies [82, 83]. In the present research, bortezomib appeared to prevent the increase in vascular permeability and the increase in tissue water content. These observations are supported by previous studies that link proteasome inhibition to reduced endothelial activation and permeability in inflammatory settings [84]. We did not directly assess the molecular markers of vascular leakage or tight junction integrity in the present study, but the reduction in Evans blue concentration in organs, tissue edema and MPO and EPO activities in treated animals indirectly supports a protective role of proteasome inhibition against vascular barrier dysfunction suggesting a stabilizing effect on endothelial integrity.

Although not directly assessed in this study, it is plausible that the proteasome inhibitor influenced cellular signaling networks such as NF-κB, which governs inflammatory responses. The NF-κB is a key driver of inflammation, its activation relies on proper proteasome function [85]. The NF-kB would be activated by the proteasome responsible for the degradation and cleavage of the IκB-NF-κB complex, the release of the factor NF-κB and its translocation into the nucleus for the expression of pro-inflammatory mediators [86].

The decrease in the rate of vascular permeability, which precedes the infiltration of inflammatory cells, may be due to the fact that the inhibition of the proteasome prior to envenomation can prevent the activation of the NF-kB factor, which could be activated by Toll-like receptors (TLR-2 and TLR-4) [73]. TLRs and their signaling pathways are currently being validated as potential immune-modulatory targets during scorpion envenomation pathogenesis [15, 17, 73]. Since these receptors are expressed in circulating and resident immune cells, including mast cells [87]. Inhibition of this signaling pathway prevents the release of preformed inflammatory mediators such as histamine and lipid derived mediators [6, 8], and thereby reduced vascular permeability. The decrease in peroxidase activities (MPO and EPO) in peripheral tissues after Aah envenomation in the presence of bortezomib could be partly attributed to the reduced vascular permeability observed in this study, which may have limited leukocyte extravasation. However, this reduction might also result from additional mechanisms, such as the apoptotic effects of proteasome inhibition on leukocytic cells, which may contribute to their immunosuppression [88-90]. It could also contribute to the reduction of the involved inflammatory mediators in the recruitment of polymorphonuclear cells such as cytokines (IL-2, IL-5, IL-8) [91] and adhesion molecules on the endothelium like E selectins, VCAM -1 and ICAM-1 and P-selectin [92-94]. 

The inhibition of proteasome resulted also in the prevention of oxidative balance alteration during Aah envenomation. This could be explained by the reduction in infiltrated neutrophil and eosinophil cells which represent important sources of reactive oxygen intermediates such as H_2_O_2_ and NO [66, 67]. Previous studies demonstrated also that increased production of ROS leads to the activation of NFĸB. This, in turn, stimulates the synthesis of other pro-inflammatory mediators such as adhesion molecules, which contribute to exacerbation of the inflammatory response [95]. It has been earlier shown that the inhibition of the proteasome system with the use of selective inhibitors blocks the induction of iNOS by preventing activation of nuclear factor‐κB [96-98]. This is associated with the upregulation of several endogenous antioxidants such as SOD, GSH‐Px and catalase contributing to significant improvement of tissue alteration [99]. This was in accordance with our results and with another previous report, which demonstrated that proteasome system inhibition may lead to increased antioxidant enzyme activities [100]. It reduces tissue oxidative damage which in turn prevents the release of the enzymatic cell contents (CPK, LDH, ALP, and ALT), as well as urea and creatinine levels. 

The mechanism of the anti-inflammatory effect observed following inhibition of the proteasome prior to envenomation of mice might be more complex. Based on the results obtained in this study and supported by bibliographic data, we suggest that proteins modified by conjugation with lipid peroxidation products produced during scorpion envenomation may undergo ubiquitination and subsequent degradation by the proteasome [101, 102]. These degraded proteins can then be presented to T cells *via* a class 1 MHC molecule. The onset of a cytotoxic immune response can worsen the tissue damage observed after scorpion envenomation. According to these data, it is possible to suggest that during scorpion envenomation, tissue damage is mediated by both the Th2 and Th1 immune pathways involving the proteasome system [11].

## Conclusion

In conclusion, the results of this study demonstrate, for the first time, the immunomodulatory role of the proteasome during scorpion envenomation. Administration of bortezomib effectively suppresses systemic inflammation by reducing vascular permeability, tissue edema, and peroxidase activities (MPO and EPO), while also enhancing the expression of antioxidants such as GSH and catalase. These effects ultimately contribute to the prevention of lipid peroxidation and tissue damage. Further investigations are warranted to explore the involvement of key inflammatory pathways, including NF-κB and Nrf2 signaling, as well as to elucidate the precise mechanisms underlying the proteasome-mediated regulation of inflammation and oxidative stress.

### Abbreviations

Aah: *Androctonus australis hector*, ALP: alkaline phosphatase, ALT: alanine aminotransferase, ARE: antioxidant response element, CAT: catalase, COX-2: cyclooxygenase 2, CPK: creatine-phosphokinase, CRP: C-reactive protein, DTNB: 5,5'-dithiobis(2-nitrobenzoic acid), EDTA: ethylenediaminetetraacetic acid, EPO: eosinophil peroxidase, GPx: glutathione peroxidase, GSH: reduced glutathione, ICAM-1: intercellular adhesion molecule 1, iNOS: inducible nitric oxide synthase, LDH: lactate dehydrogenase, MDA: malondialdehyde, MHC: major histocompatibility complex, MPO: myeloperoxidase, NF-kB: nuclear factor kappa B, NMRI: Naval Medical Research Institute, NRf2: nuclear erythroid-2 like factor-2, OPD: o-phenylenediamine, PBS: phosphate-buffered saline, SDS: sodium dodecyl sulfate, SEM: standard error of the mean, TCA: trichloroacetic acid, TLRs: toll-like receptors, VCAM-1: vascular cell adhesion molecule 1.

## Data Availability

The datasets used and/or analyzed during the current study are available from the corresponding author on reasonable request.
